# A Case of Small Cell Carcinoma of Bladder With Significant Tumor Reduction Following Combination Chemotherapy and Radiation Therapy

**DOI:** 10.1002/iju5.70031

**Published:** 2025-04-14

**Authors:** Rintaro Yoshitake, Syunsuke Taguchi, Yoshimasa Harada

**Affiliations:** ^1^ Nagahama Red Cross Hospital Nagahama Japan; ^2^ Hikone Municipal Hospital Hikone Japan

**Keywords:** carboplatin, etoposide, radiation, small cell carcinoma, urinary bladder

## Abstract

**Introduction:**

Small cell carcinoma of the bladder (SCCB) is a rare and aggressive disease with no established standard treatment. We report a case of SCCB that initially progressed with chemotherapy but responded well to a combination of chemotherapy and radiotherapy.

**Case Presentation:**

A 62‐year‐old man presented with painless gross hematuria. Transurethral resection of the bladder tumor (TURBT) confirmed SCCB. Gemcitabine‐carboplatin (GEM‐CBDCA) and pembrolizumab were ineffective, leading to rapid tumor progression. The patient developed bladder tamponade and obstructive renal failure. Emergency radiotherapy (30 Gy) controlled bleeding, and etoposide‐carboplatin chemotherapy resulted in significant tumor shrinkage.

**Conclusion:**

This case highlights the potential effectiveness of etoposide‐carboplatin and radiotherapy in SCCB, especially in cases resistant to other treatments. Further studies are needed to establish optimal treatment strategies for SCCB.

**Summary**

- A 62‐year‐old man with small cell carcinoma of the bladder had rapid tumor growth after initial treatment.
- Emergency radiation therapy and carboplatin‐etoposide chemotherapy then resulted in significant tumor shrinkage, showing that this combination can be effective.

## 1 Introduction

SCCB is a rare and aggressive malignancy, with no established standard treatment. We report a case of SCCB that initially showed significant tumor progression despite standard urothelial carcinoma treatments but subsequently responded remarkably to chemotherapy and radiotherapy, following a treatment strategy similar to that used for small cell lung cancer (SCLC).

## 2 Case Presentation

### 2.1 Patient Information

A 62‐year‐old male presented with asymptomatic macroscopic hematuria. His past medical history included hypertension and hyperuricemia. He had a 10‐year history of smoking 20 cigarettes per day, which he discontinued at the age of 30.

**FIGURE 1:**
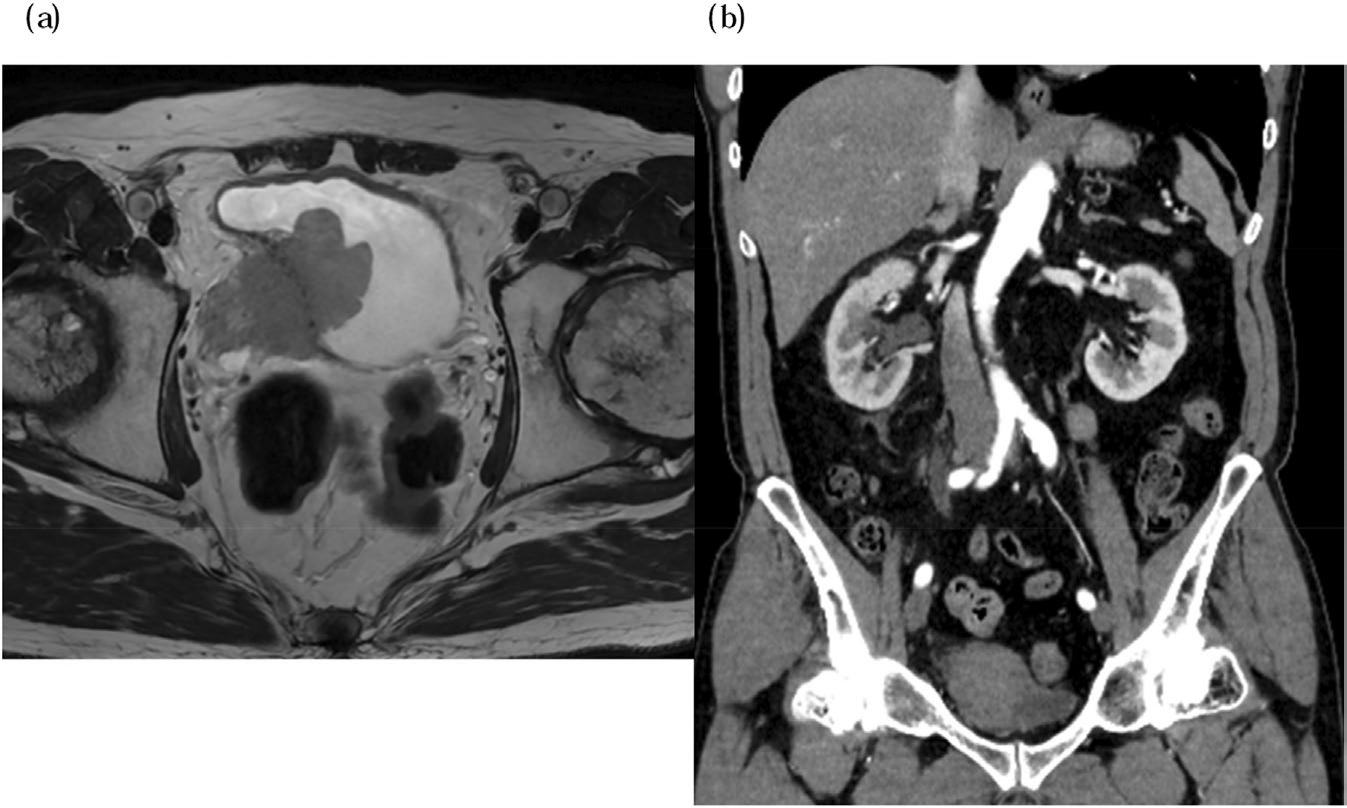
(a) The MRI findings reveal a 5 cm mass located on the right wall of the bladder. There is suspicion of infiltration into the pelvic wall, and infiltration is also noted at the right ureteral orifice. (b) The contrast‐enhanced chest and abdominal CT findings show no evidence of distant metastasis from the bladder tumor. Additionally, the right kidney exhibits hydronephrosis graded as SFU grade 2.

**FIGURE 2:**
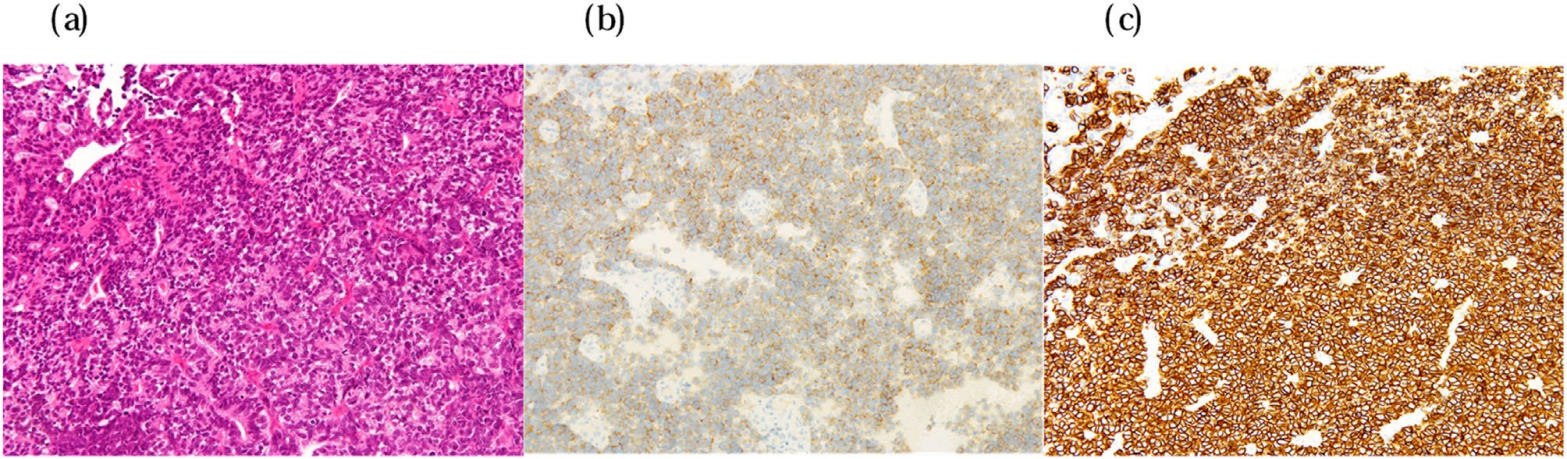
The pathological findings of the TUR specimen showed that approximately 80% was small cell carcinoma, with the remaining portion consisting of UC components. Immunohistochemistry revealed GATA3‐positive atypical cells at the edges, but most of the atypical cell clusters were negative for chromogranin A, weakly positive for synaptophysin, and diffusely positive for CD56. (a) Hematoxylin–eosin stain. (b) Immunohistochemical staining for synaptophysin. (c) Immunohistochemical staining for CD56.

Magnetic resonance imaging (MRI) revealed a 5 cm tumor on the right lateral wall of the bladder, invading beyond the bladder wall (Figure 1a). Computed tomography (CT) revealed grade 2 hydronephrosis (SFU classification) on the right kidney but no evidence of lymph node involvement or distant metastasis (Figure 1b). Urine cytology was classified as class V. TURBT was performed, and histopathology confirmed invasive urothelial carcinoma with a small cell carcinoma component (Figure 2). The clinical diagnosis was SCCB cT4bN0M0. The pre‐treatment Neuron‐specific enolase (NSE) level was 37.9 ng/mL.

**FIGURE 3:**
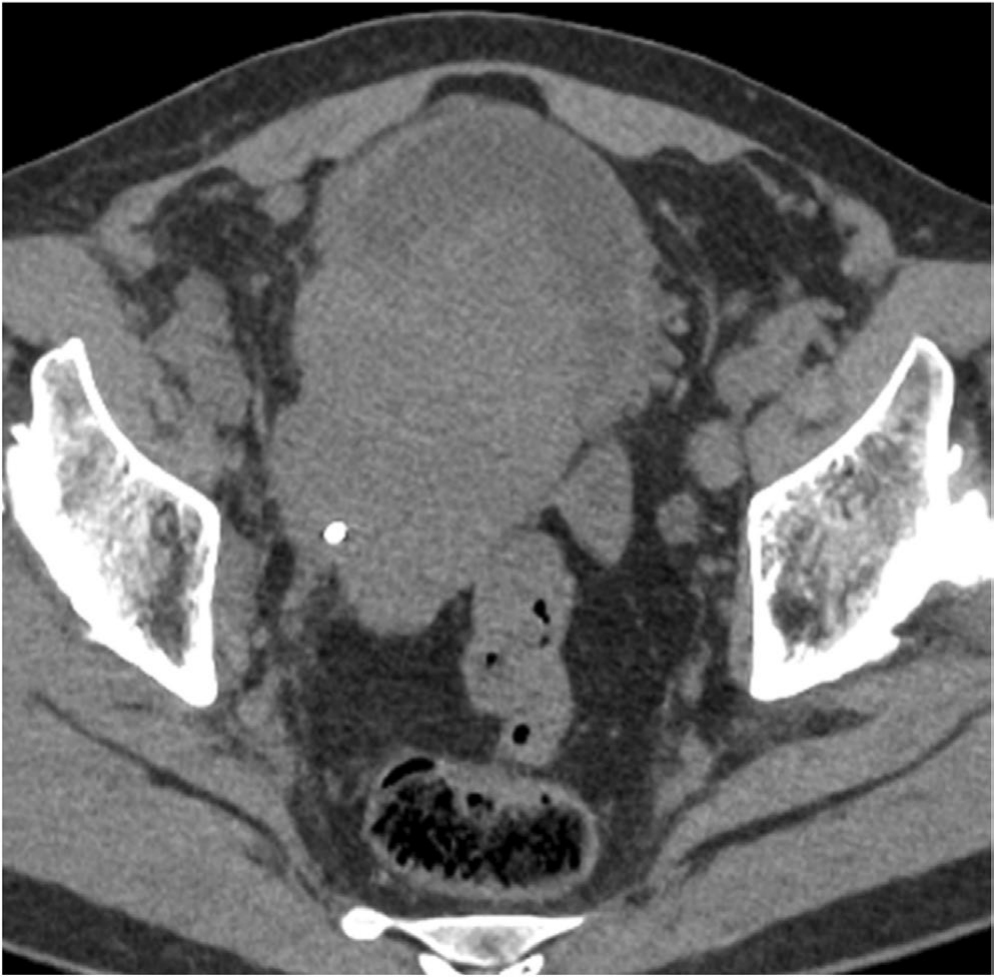
The chest and abdominal CT findings indicate that the bladder tumor has significantly increased in size, with evidence of bilateral hydronephrosis and a metastasis to the right rib.

Due to the lack of established treatment guidelines for SCCB, we initially administered 2 cycles of GEM‐CBDCA, a standard regimen for urothelial carcinoma. However, the tumor showed no significant shrinkage, and the response was classified as stable disease (SD). We then administered 2 cycles of pembrolizumab, but the tumor rapidly progressed (Figure 3), leading to bilateral hydronephrosis and right rib metastasis. NSE increased to 1030 ng/mL, and Progastrin‐releasing peptide (ProGRP) was elevated at 129 pg/mL. The patient developed bladder tamponade and post‐renal acute kidney injury, necessitating hospitalization.

**FIGURE 4:**
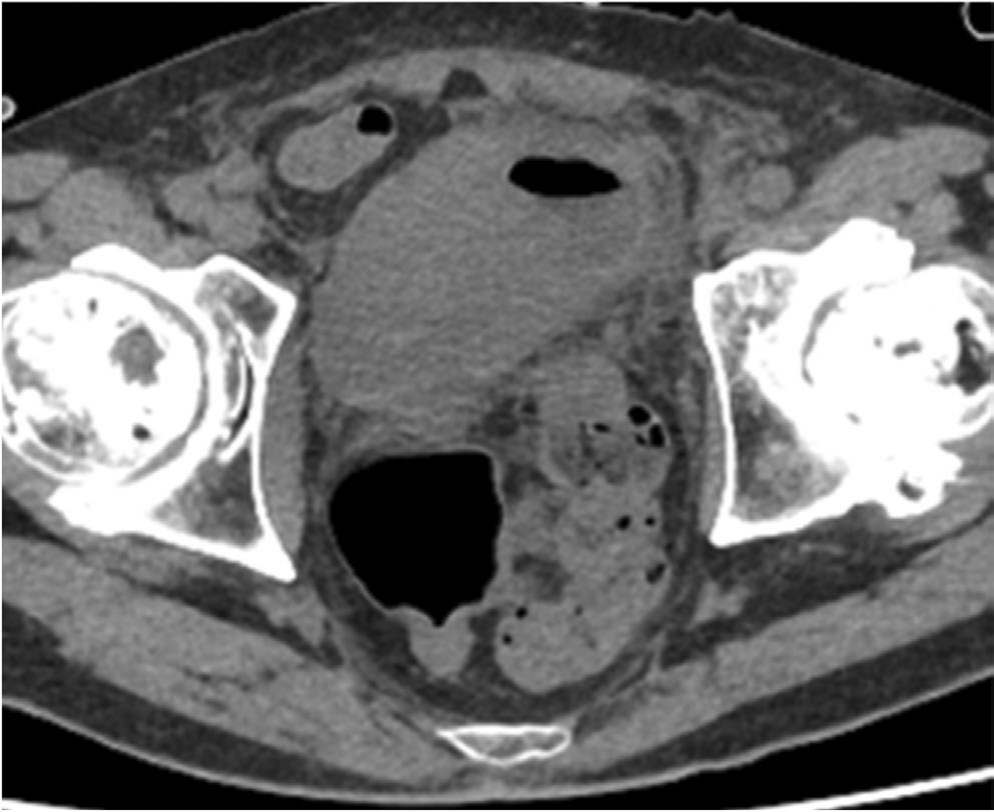
The chest and abdominal CT findings reveal a significant reduction in the size of the bladder tumor.

Upon admission, the patient underwent bilateral nephrostomy, which resulted in improved renal function. However, persistent hemorrhagic cystitis required continuous bladder irrigation and frequent transfusions. In consultation with radiation oncologists, we initiated hemostatic radiotherapy (30 Gy/10 fractions), successfully controlling bleeding. Subsequently, chemotherapy following the standard SCLC regimen, carboplatin and etoposide (CE), was introduced at a 90% dose. Despite developing febrile neutropenia and *Clostridioides difficile* colitis during the first cycle, he achieved significant tumor shrinkage. After the second cycle, further tumor regression was observed (Figure 4). Regarding tumor markers, NSE decreased to 156 ng/mL and ProGRP to 51.6 pg/mL after 1 cycle. After 2 cycles, NSE further decreased to 50.0 ng/mL and ProGRP to 46.0 pg/mL. The patient was discharged and later transferred to another hospital due to relocation.

## 3 Discussion

SCCB is an uncommon histological variant of bladder cancer, accounting for approximately 0.7% of all bladder tumors [1]. It shares pathological characteristics with SCLC, including poorly differentiated small cells with scant cytoplasm, hyperchromatic nuclei, and a high mitotic rate. Due to its aggressive nature, SCCB is often diagnosed at an advanced stage, leading to a poor prognosis [2]. According to Choong et al., the 5‐year survival rates for stage II, III, and IV SCCB are 63.6%, 15.4%, and 10.5%, respectively [3].

Although treatment strategies for SCCB are not well established, its pathological similarity to SCLC has led to the frequent adoption of chemotherapy regimens used for SCLC, such as platinum‐based therapies (cisplatin/etoposide or carboplatin/etoposide). The National Comprehensive Cancer Network (NCCN) guidelines recommend neoadjuvant chemotherapy followed by local therapy (radical cystectomy or radiotherapy) for resectable tumors and systemic chemotherapy for metastatic cases [4]. However, some studies suggest that SCCB may respond to therapies used for urothelial carcinoma. Reza et al. reported a case successfully treated with gemcitabine and cisplatin, achieving a median survival of 42.4 months [5]. Additionally, Hatayama et al. described a case where pembrolizumab achieved a complete response after disease recurrence [6].

In our case, we initially treated the patient with GEM‐CBDCA, a standard regimen for conventional urothelial carcinoma. The patient had hoped to undergo curative treatment, such as radical cystectomy, if chemotherapy was effective. However, the tumor showed little reduction in size. Therefore, we switched to pembrolizumab as a second‐line therapy, but this treatment also failed to control tumor progression. As a third‐line therapy, we administered the CE regimen, which resulted in a favorable response. This outcome supports the notion that SCCB may respond better to SCLC‐directed chemotherapy. Additionally, etoposide‐related adverse events, including febrile neutropenia and *C. difficile* colitis, were managed without life‐threatening complications, suggesting that CE therapy can be safely administered with appropriate supportive care.

Radiotherapy played a crucial role in this case. In SCLC, combined chemoradiotherapy has been shown to improve overall survival compared to chemotherapy alone [7]. A meta‐analysis by Pignon et al. demonstrated a 14% reduction in mortality risk and a 5.4% improvement in 3‐year survival when thoracic radiotherapy was added to chemotherapy [8]. Similarly, studies on SCCB suggest a potential benefit of radiotherapy in combination with chemotherapy. Karpman et al. reported a 70% survival rate at a median follow‐up of 34 months in patients treated with chemoradiotherapy [9], and Akamatsu et al. found that the 3‐year survival rate in SCCB patients receiving both chemotherapy and radiotherapy was 50%, compared to a significantly lower rate in those receiving radiotherapy alone [10]. The marked tumor shrinkage observed in our patient after CE therapy may have been enhanced by prior radiotherapy, highlighting the importance of multimodal treatment strategies. Additionally, a recent report by Mita et al. described a case of localized SCCB treated with chemotherapy and radical cystectomy. After recurrence in the abdominal lymph nodes, additional chemotherapy and immune checkpoint inhibitors (ICI) were administered, and the metastatic lesions eventually shrank following radiotherapy [11]. This suggests that SCCB may require consideration of multidisciplinary treatment approaches.

Given the rarity of SCCB, accumulating clinical data is essential to establish optimal treatment approaches. While our case suggests that chemotherapy regimens for SCLC and the integration of radiotherapy may improve outcomes, further studies are required to validate these findings.

## 4 Conclusion

We reported a case of SCCB that initially progressed despite urothelial carcinoma‐directed therapy but responded well to chemoradiotherapy following an SCLC‐based treatment strategy. This case underscores the potential efficacy of CE therapy and radiotherapy in SCCB and highlights the need for further clinical evidence to inform treatment strategies.

## Consent

The authors have nothing to report.

## Conflicts of Interest

The authors declare no conflicts of interest.
